# Facilitators and barriers to routine intimate partner violence screening in antenatal care settings in Uganda

**DOI:** 10.1186/s12913-022-07669-0

**Published:** 2022-03-02

**Authors:** Ronald Anguzu, Laura D. Cassidy, Kirsten M. M. Beyer, Harriet M. Babikako, Rebekah J. Walker, Julia Dickson-Gomez

**Affiliations:** 1 Division of Epidemiology and Social Sciences, Institute for Health and Equity, Medical College of Wisconsin, 8701 Watertown Plank Road, Milwaukee, Wisconsin USA; 2 Department of Epidemiology and Biostatistics, Makerere University School of Public Health, Makerere University College of Health Sciences, New Mulago Gate Road, Mulago, Kampala, Uganda; 3 Department of Child Health and Development Center, School of Medicine, Makerere University, College of Health Sciences, Mulago Hill, Hospital Complex, P.O. Box 7072, Kampala, Kampala, Uganda; 4 Center for Advancing Population Sciences (CAPS), Medical College of Wisconsin, Milwaukee, Wisconsin USA; 5 Division of General Internal Medicine, Medical College of Wisconsin, Milwaukee, Wisconsin USA

**Keywords:** Intimate partner violence screening, Antenatal care services, Barriers and Facilitators, Health service delivery

## Abstract

**Background:**

Uganda clinical guidelines recommend routine screening of pregnant women for intimate partner violence (IPV) during antenatal care (ANC). Healthcare providers play a critical role in identifying IPV during pregnancy in ANC clinics. This study explored facilitators and barriers for IPV screening during pregnancy (perinatal IPV screening) by ANC-based healthcare workers in Uganda.

**Methods:**

We conducted qualitative in-depth interviews among twenty-eight purposively selected healthcare providers in one rural and an urban-based ANC health center in Eastern and Central Uganda respectively. Barriers and facilitators to IPV screening during ANC were identified iteratively using inductive-deductive thematic analysis.

**Results:**

Participants had provided ANC services for a median (IQR) duration of 4.0 (0.1–19) years. Out of 28 healthcare providers, 11 routinely screened women attending ANC clinics for IPV and 10 had received IPV-related training. Barriers to routine IPV screening included limited staffing and space resources, lack of comprehensive gender-based violence (GBV) training and provider unawareness of the extent of IPV during pregnancy. Facilitators were availability of GBV protocols and providers who were aware of IPV (or GBV) tools tended to use them to routinely screen for IPV. Healthcare workers reported the need to establish patient trust and a safe ANC clinic environment for disclosure to occur. ANC clinicians suggested creation of opportunities for triage-level screening and modification of patients’ ANC cards used to document women’s medical history. Some providers expressed concerns of safety or retaliatory abuse if perpetrating partners were to see reported abuse.

**Conclusions:**

Our findings can inform efforts to strengthen GBV interventions focused on increasing routine perinatal IPV screening by ANC-based clinicians. Implementation of initiatives to increase routine perinatal IPV screening should focus on task sharing, increasing comprehensive IPV training opportunities, including raising awareness of IPV severity, trauma-informed care and building trusting patient-physician relationships.

**Supplementary Information:**

The online version contains supplementary material available at 10.1186/s12913-022-07669-0.

## Background

Intimate partner violence (IPV) is a pervasive yet preventable global health problem which refers to actual or threatened abuse by an intimate partner that may be physical, sexual, psychological, or emotional in nature [1]. In Uganda, IPV rates among women of reproductive age (15–49 years) are high at 29.3%, 22.5%, and 16.6% for psychological, physical and emotional IPV respectively [2] while, 10.6% of women experience IPV during pregnancy (perinatal IPV) [2]. Regional differences show a higher IPV prevalence among pregnant rural residents (11.4%) compared to their urban counterparts (8%) [3]. Exposure to IPV during pregnancy increases the risk for low birth weight babies, premature rupture of membranes [4], induced abortions [5, 6], HIV acquisition [7, 8], IPV-related injury [3] and disability [9, 10]. Addressing IPV merits the involvement of healthcare systems to detect and prevent morbidity and mortality associated with IPV among vulnerable populations, including perinatal IPV survivors [11, 12]. Evidence shows that IPV screening effectively reduces depressive symptoms and improves some pregnancy outcomes [13]. Implementation of effective programs are essential in order to routinely detect and respond to IPV during pregnancy [14]. Antenatal care (ANC) clinics present an opportune setting to screen for perinatal IPV in women seeking ANC services.

IPV is a form of gender-based violence (GBV) in which abusive behavior is perpetrated towards survivors based on differences in power dynamics within an intimate relationship [15]. In this paper, we use IPV except for cases in which the larger concept of GBV is more appropriate, such as the GBV training given to healthcare providers, which includes detecting and responding to all forms of GBV such as sexual assault against strangers or when used by participants in quotations.

The World Health Organization (WHO) recommends that pregnant women attend a minimum of eight ANC visits in order to optimize maternal, fetal and newborn health outcomes [16], which includes routine IPV screening in ANC clinics. The Uganda Clinical Guidelines (UCG) recommends IPV screening in healthcare settings to be conducted comprehensively alongside screening for chronic medical conditions such as hypertension during pregnancy (pre-eclampsia), and gestational diabetes among others [17]. These WHO and UCG recommendations are promising public health interventions that may reduce the occurrence of adverse maternal and child health outcomes due to timely detection of risk factors in early gestation. The process of provision of routine ANC services in Uganda involves triaging, group health education, consultation that includes detailed history taking, physical examination, conducting diagnostic laboratory or radiological testing, whole management involves providing any needed medical treatment and appropriate referrals. Provider administered IPV screening may be provided along this continuum of care specifically during triage and clinical consultations. Although healthcare provider screening, counseling and management of IPV during ANC are prevention efforts recommended to be conducted within healthcare settings [7, 17, 18], anecdotal evidence suggests that during patient-physician assessments, ANC providers usually focus on obstetric risk screening only. Such clinical practices may increase the likelihood of perinatal IPV going undetected and untreated due to overlooking subtle or overt signs related to IPV.

Several studies that describe the challenges as well as the facilitators for IPV screening by healthcare workers have been conducted in high income countries (HIC) [19–22]. Some barriers to IPV screening from studies conducted in HIC included lack of provider training on IPV, few healthcare workers who receive specialized training in IPV, time constraints, inadequate privacy during clinical assessments as well as poor employer support of healthcare providers [23]. Due to the complexity of risk factors and prevention strategies for IPV, systems-level interventions have been proposed. There is paucity of evidence from Low and Middle Income Countries (LMICs) [24, 25], including Uganda, [26–29] that describes whether or how healthcare providers screen for IPV among populations of women seeking ANC services.

The National Policy on Elimination of Gender-Based Violence (GBV) of Uganda provides a policy framework aimed at addressing critical gaps in Uganda’s GBV response [30]. This includes guiding the multi-sectoral and multi-institutional implementation of comprehensive GBV prevention measures by state and non-state actors such as coordinating support and referrals between health, social, and law enforcement sectors. Screening for IPV in healthcare settings presents unique challenges due to the socio-cultural context within which IPV is perpetrated and condoned. Women may be deterred from disclosing IPV because of traditional practices such as bride price, which reduces women’s independence in decision making to seek supportive care for perinatal IPV [31]. In fact, referral of IPV survivors by healthcare providers and Village Health Teams is low partly due to low IPV identification [3]. However, while many women do not seek help after experiencing IPV, they are still more likely to seek healthcare than other supportive services. A national survey conducted in Uganda found that 10.5% of women who experienced IPV used health services following violence, 8.5% reported to local councils, 2% notified police, 0.7% sought social services and 0.2% sought legal proceedings [3]. Limited support- or care- seeking behavior following IPV exposure reduces the chances of being screened for IPV. This national survey did not assess the extent of IPV disclosure among care seeking IPV survivors in Uganda. However, a mass-media, ‘edutainment’ experiment aimed at changing norms and behaviors in one rural community in Uganda showed an increase in willingness to disclose IPV despite not reducing their acceptance of IPV [32]. Results from a randomized clinical trial to increase IPV screening in healthcare settings were inconclusive [33]. It is imperative that perinatal IPV is prioritized as a critical public health challenge that necessitates commitment and multi-institutional responses at national, health facility and community levels.

An in-depth understanding of the challenges and enablers experienced by health facility-based providers to IPV screening using multi-level frameworks is essential in order to advance the roles healthcare providers play in implementation of IPV prevention and response strategies. In order to address this knowledge gap, our study examined facilitators and barriers to IPV screening by healthcare providers during ANC in rural and urban Uganda.

## Methods

### Study design and setting

This health facility-based, qualitative study was conducted among twenty-eight healthcare providers in two public health facilities in rural and urban settings of Uganda. One study site is Luuka district, a predominantly rural area located in the Busoga sub-region of Eastern Uganda with a total population of 238,020 [34]. Luuka district is mostly engaged in agricultural activities, specifically growing sugarcane as their main commercial activity. The other study site is Kisenyi division, an administrative area of Kampala Capital City Authority (KCCA), located in the central sub-region of Uganda with a population of 338,665 or (20.4%) of the total KCCA population [34]. Kisenyi is predominantly urban with industrialized areas and several informal (slum) settlements. Informal housing settlements are areas of unplanned, unauthorized settlements where housing units are constructed on land occupied illegally or where the occupants have no legal claim and the housing not in compliance with building regulations [35].

### Sample and recruitment

Two health facilities at health center IV (HCIV) level were purposively selected from Luuka district and Kisenyi division of KCCA in accordance with the hierarchy of Uganda’s health referral system because HCIVs provide comprehensive emergency obstetric and neonatal care (CEmONC) services [36]. These CEmONC services include the following signal functions at health facilities: (i) performing surgery such as caesarian sections and providing blood transfusions in addition to providing basic Emergency Obstetric and Neonatal Care [37]. Kiyunga HCIV is the main referral healthcare facility in Luuka district while Kisenyi HCIV has the highest patient turnover among HCIVs in KCCA. These two healthcare facilities were purposively selected because one was rural (Kiyunga HCIV) and the other urban (Kisenyi HCIV) in addition to potential differences in barriers and facilitators to routine IPV screening.

The daily duty rosters were used to purposively select healthcare providers in ANC clinics. The first author and interviewer introduced the study to the health facility in-charge and clinicians either in-person or through phone calls to introduce the study and establish the providers’ willingness to participate. Participants were told that their decision to participate was voluntary and was not shared with supervisors. Willing participants chose convenient dates, times and locations within ANC clinics for the interviews. Rooms in ANC clinics for interviews were private and without interruption. On the interview day, written informed consent was obtained before data collection in which confidentiality and privacy were upheld. The interviewer presented letters of ethical clearance from The AIDS Support Organization (TASO) IRB, Medical College of Wisconsin (MCW) IRB and Uganda National Council of Science and Technology (UNCST) and letters of administrative permission from the Director of Public and Environmental Health at KCCA and the District Health Officer of Luuka district in-charges of ANC units in order to conduct interviews in the respective selected district health facilities.

The twenty-eight licensed healthcare workers that were initially contacted and accepted to participate were purposively selected to reflect a range of experiences and clinical training to provide ANC services including obstetric and gynecological care, health education and promotion of reproductive health. Data were collected in a period of ten days in Luuka district and fifteen days in Kisenyi, Kampala. The Daily duty rota contained a list of all on-call providers in ANC each week, therefore providing the sampling frame from which to identify, introduce the study to (physically or by phone) and invite providers approached to participate in the study. After introducing the study, participants were asked to take a few days to think about participating before making their decision. Interviews stopped when the interviewer observed that no new information was being reported; hence saturation was achieved at 28 respondents. All participants approached were willing and participated in the study. These healthcare providers included obstetricians and gynecologists, general physicians/medical officers, and midwives. Certificate- and diploma-level midwives who provided ANC services were interviewed. Certificate-level midwives are healthcare providers, referred to as enrolled midwives, who receive one-year professional midwifery training. Diploma-level midwives, referred to as Assistant Nursing Officers, are those who received two-year midwifery training. Two Obstetricians/Gynecologists (OBGYN) were recruited from one purposively selected national referral hospital to ensure credibility of our findings from the sampled population.

### Data collection procedure

To investigate the facilitators and barriers to healthcare provider screening for IPV among pregnant women during ANC, we conducted in-depth interviews. All interviews were conducted in English. In-depth interviews were audio recorded and lasted an average of 60 min. Interviews were conducted by RA who is trained and experienced in collecting qualitative data. Field notes were taken during or immediately after each interview. The in-depth interview guide contained questions and probes about clinical practice and experiences of ANC services offered from the provider’s perspective.

### In-depth interview guide

The multi-level framework by Chaudoir and colleagues [38], guided development of the in-depth interview guide. We pre-tested our in-depth interview guide on three respondents in one urban based ANC facility other than the two selected health units. The in-depth interview guide asked questions that were categorized into provider-, partner-, and organizational-related characteristics and referral practices. Questions about provider-related characteristics included healthcare worker training, clinic workload, work hours and competing priorities. Our in-depth interview guide also probed providers’ attitudes towards IPV survivors and feelings about their vulnerability. Questions about patient characteristics from the providers’ perspectives included whether IPV survivors disclose IPV or not and potential reasons why. We probed for whether providers routinely or consistently screen for IPV during each ANC visit and the extent of IPV screening and referral practices. Generally, routine IPV screening varies in definition ranging from screening at every visit, every first ANC visit or every annual exam [39]. However, for this study, we defined routine IPV screening as screening for IPV at every ANC visit. In addition, timing of women’s first ANC visit, male partner ANC attendance and the burden of perinatal IPV among women attending ANC were explored.

First, our in-depth interview guide asked providers what prompts their suspicion of IPV. We asked for suggestions or recommendations on how to increase providers’ identification of IPV during pregnancy. Participants were asked to narrate their personal experiences and the training they received for managing perinatal IPV survivors. Secondly, our in-depth interview guide also included questions regarding whether referrals for specialist services for IPV and follow-ups are made. In addition, we asked providers about any factors that facilitate or impede routine IPV screening by providers in ANC clinics. We also probed for whether gaps exist between usual screening practices for IPV by clinicians and recommendations from UCG [17]. Thirdly, partner-related probes explored characteristics of women’s partners that may impede women from disclosing IPV. In addition, we elicited suggestions from providers on how to improve IPV disclosure to healthcare workers as well as how the safety of perinatal IPV survivors may be addressed. Fourthly, we probed for organizational factors, including the availability and extent to which clinical guidelines on IPV screening and referral were implemented, the availability of IPV screening tools and clinic scheduling.

### Qualitative data analysis

All interviews were transcribed verbatim. Transcripts were then analyzed using MAXQDA® a qualitative data analysis software [40]. The transcripts were read and re-read in a staged, iterative process in order to identify emerging codes by two study team members. These two coders were RA, a doctoral candidate in public and community health, and licensed general medical doctor in Uganda and JDG an experienced anthropologist and global health researcher at the Institute for Health and Equity, MCW.

**Fig. 1:**
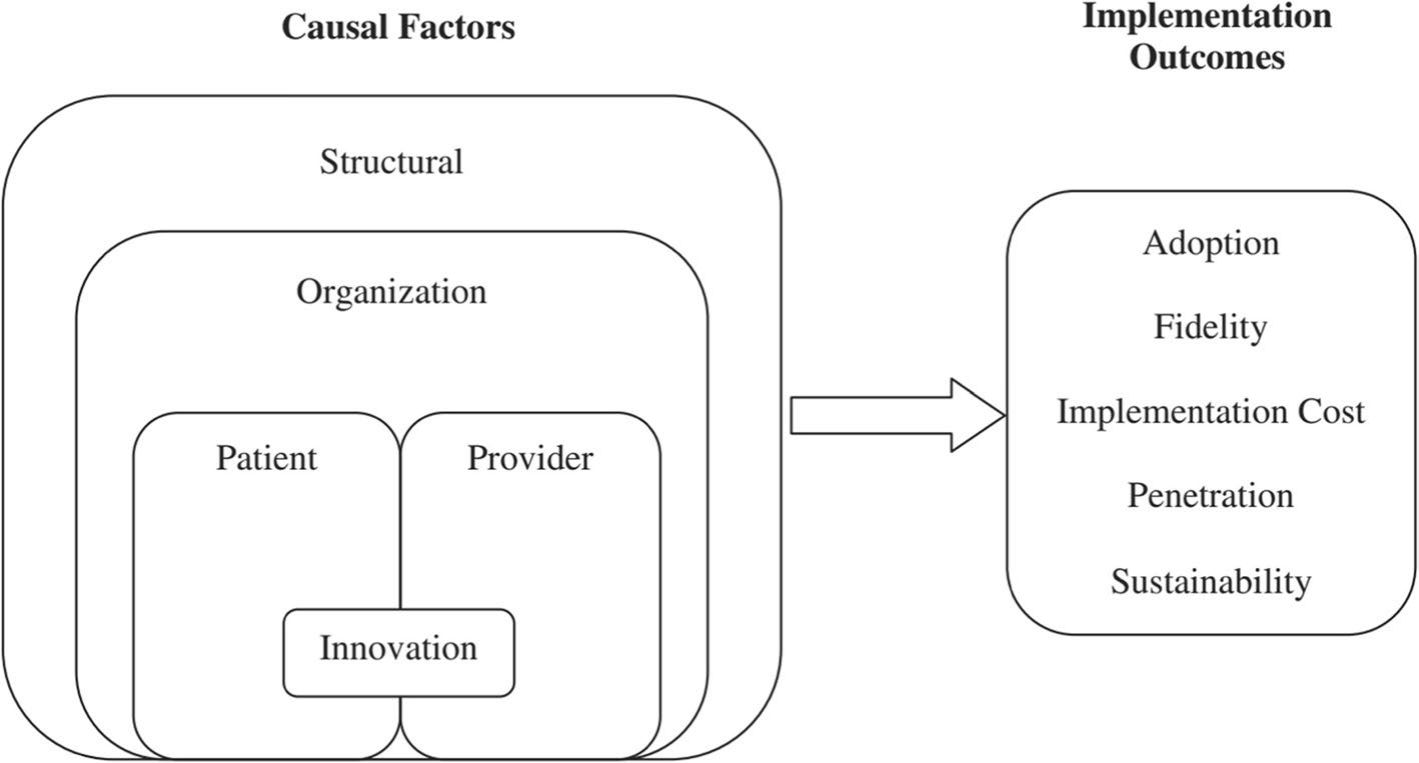
Multi-level framework predicting implementation outcomes adapted from Chaudoir and colleagues (2013)

In the initial phase of analysis, themes determined a priori were adapted from the multi-level framework to predict implementation outcomes developed by Chaudoir and colleagues [38], as shown in Fig. 1. This comprehensive multi-level framework posited five factors namely, structural, organizational, provider, patient, and innovation level factors that may influence implementation outcomes [38], including routine IPV screening by healthcare providers.

Secondly, each of the five themes determined a priori were then sub-categorized into barriers and facilitators to IPV screening during ANC. Thirdly, four transcripts (20% of all transcripts) were read by each of the two coders to identify codes regarding barriers or facilitators to IPV screening during ANC. Fourth, consensus was reached on all emergent codes after discussions between the two coders about code names and code definitions. We then collaboratively developed a coding tree. This was conducted using an iterative inductive-deductive thematic approach between coders. Fifth, comparisons of statements were made to identify differences and similarities between rural and urban-based ANC providers, and provider specialty.

## Results

### Screening practices for IPV

As described in Table 1, twenty-two ANC providers were women, six were men while four ANC providers were general physicians, eight assistant nursing officers, and fourteen enrolled midwives. This study identified barriers that affect routine IPV screening and facilitators that improve routine IPV screening in two ANC clinics in rural and urban Uganda. Four emergent themes and quotes that illustrate barriers and facilitators to routine IPV screening are described in Table 2. Out of all twenty-eight healthcare providers, eleven reported conducting routine screening for IPV among women attending ANC clinics, while ten reported having received IPV related training. Among the eleven healthcare providers who conducted routine IPV screening, seven were urban–based ANC providers, while four were based in ANC clinics in rural settings. Among all ten healthcare providers who received IPV related training, only three were rural-based ANC providers compared to seven who were urban-based ANC service providers. One healthcare provider acknowledged not routinely screening for IPV and instead relied on women to initiate disclosure as explained by the following excerpt:

“We don’t routinely screen for intimate partner violence unless a patient volunteers that information, but we don’t go into it so much.” (#4, M, OBGYN, Urban).

**Table 1:** Participant characteristics and provider screening practices for intimate partner violence in antenatal clinics

	**Overall, *N* = 28**	**Urban, *N* = 15**	**Rural, *N* = 13**
**Characteristic**		**n**	n
Duration of clinical practice, median (IQR) years	4.0 (0.1–19)	5.0 (1.3–15)	2.0 (0.1–19)
Gender			
Male	7	3	4
Female	21	12	9
Clinical discipline			
Enrolled midwife	14	7	7
Registered midwife	8	5	3
Medical officer^a^	4	1	3
OBGY^b^	2	2	0
ANC setting			
Urban	15	-	-
Rural	13	-	-
Routine IPV screening			
No	17	8	9
Yes	11	7	4
IPV training			
No	18	8	10
Yes	10	7	3

^a^General medical practitioner, ^b^Obstetrics and Gynaecology

**Table 2:** Emergent themes and sub-themes

	**Emergent themes**	**Sub-themes**
1	Resources	Staffing shortages
		Lack of places to refer women facing IPV
		Availability of GBV screening tools
		Modification of ANC cards by including IPV items
		Inadequate physical space
2	Awareness and training	Lack of comprehensive IPV training
		Inadequate IPV screening knowledge
		Need for brief IPV screening tools
3	Lack of awareness of IPV severity	Provider misperceptions
4	Establishing trust	Provider initiated probing
		Rapport building
		Patient initiated disclosure

Another rural-based provider who also reported not routinely screening for IPV attributed not screening to the lack of special GBV clinics as described below:

I: So, do health workers in this clinic routinely screen women for gender-based violence?

R: No, unless one has disclosed to you that is when you can talk and help her.

I: Are there any other reasons why you think it is not routinely screened for?

R: Maybe because they don’t tell us, or maybe because we do not have a special clinic for that

(#11, F, Enrolled Midwife, Rural)

Some healthcare providers did not routinely screen for IPV because they assumed that IPV was absent in communities as explained in the following excerpt:

*“We do not ask women about gender-based violence. We assume it is not there in the community and we feel it is not there. That is what we capture in our tool and that is what we forward, but in actual sense we do not ask about gender-based violence when women come for antenatal”. *(#12, M, Medical Officer, Rural)

However, other healthcare providers reported conducting routine IPV screening during ANC clinics. This practice was exemplified by one rural-based midwife stating that:

“*It is routine because antenatal is done every day. Remember, when you are registering the mothers who have come, you ask, that is when you will have an answer of what to put in the register”. *(#1, F, Enrolled midwife, Rural)

The emergent themes that describe barriers and facilitators to routine IPV screening in ANC were: 1) Resources; 2) Awareness and training to screen and manage IPV; 3) Lack of awareness of IPV severity; and 4) Establishing trust (Table 2).

#### Resources

Identified resource-related barriers to routine IPV screening included staffing levels, ANC cards without IPV questions, and inadequate space in ANC clinics. However, many healthcare providers overcame these barriers through making referrals to specialists, modifying clinic scheduling, counseling IPV survivors, establishing special GBV units, modifying the ANC card to include IPV items and task shifting.

##### *Staffing resources*

Both rural and urban-based healthcare providers reported having limited time to listen to patients. Screening for IPV requires time to build trusting relationships in which women are comfortable disclosing IPV.

*We need to give these mothers time. When you give a mother time, she will always tell you what is happening to her. So maybe you need to give adequate time to a mother but that may not happen due to high numbers. Sometimes you have so many mothers around and of course you have to do something. *(#20, F, Enrolled midwife, Urban)

If IPV is disclosed, healthcare providers must spend more time counseling women.

“*We have little time for patients and services are affected in detecting GBV cases because if you are to detect a GBV, you have to take time with this patient talking to her, counseling her, then she will come out. *(#7, F, Assistant Nursing Officer, Rural)

Sometimes providers give preference to obstetric and fetal wellbeing often viewing psycho-social issues such as IPV as non-medical emergencies. Some healthcare providers also perceived IPV as being potentially non-fatal to the women’s lives as explained in the following excerpt.

“*Given that we have the time issue, if not being so prominent on the antenatal card, the high volume number, the focus is more on the baby and not maternal complaints. I think even in the training in the obstetric setting that component [gender-based violence] is not so much focused on. We just leave it out. Maybe it is talked about once in a while, but the focus is on those things that kill the mother and kill the baby. That is the focus during antenatal.*” (#1, F, Enrolled Midwife, Rural)

Because providers may not have adequate time to devote to addressing IPV themselves, they used experts outside ANC clinics such as psychosocial counselors to try to help women living in that situation during IPV screening and follow-up care. Providers stressed the importance of counseling services as being an opportune pathway to identify spousal abuse. This reduces the likelihood of IPV disclosure due to inadequate communication time between providers and patients. In some cases, referrals were made to counselors to screen for IPV and thereafter manage IPV according to screening and management guidelines such as task sharing by referrals to psychosocial counsellors as stated below.

*“The reason is that we have big numbers. Sometimes you are overwhelmed with the numbers. Sometimes you have to involve counselors so instead of spending a lot of time on this mother, yet you are not going to get information, you refer this mother to a counselor because you already have a long queue to work on”.* (#19, F, Enrolled Nurse, Urban)

Once psychosocial counselors screen and identify IPV, they usually continue to offer survivor support. IPV is a human rights violation. However, some IPV survivors were not aware of that fact. Providers mentioned that psychosocial counseling can aid in raising human rights awareness among IPV survivors attending ANC clinics. The following quote demonstrates this.

“*The best way is to first counsel those mothers and teach them about their rights then hand them to some organization that handles such matters like women’s organizations. Sometimes we send them to the counselors. We have a good counselor here who works with legal aid. That is what I think is the best option for them”.* (#18, M, Medical Officer, Urban)

##### Lack of places to refer women facing IPV

Some ANC service providers stated that they are deterred from routine IPV screening because they do not have knowledge of options to manage and refer IPV survivors. These providers noted how they would rather not screen for IPV without places to refer them. This results in a missed opportunity to detect IPV among ANC attendees as one obstetrician stated:

*“If you know you are going to screen and find out that she is living in a violent relationship and you know you are going to leave it at that, then I would rather not screen”. *(#24, M, OBGYN, Urban)

We found that GBV units were located in the HIV clinic only and not in the ANC clinic of the urban-based health facility. Some urban-based participants suggested creating special IPV units within ANC clinics. One urban-based midwife reported that they have a GBV clinic and focal person available within the health facility but that it is not located within the ANC unit. Many different departments make ‘internal’ referrals for GBV management to this clinic:

*“When we receive these mothers, and we feel they really need help, we refer them to the gender-based violence department which is based at the ART [anti-retroviral therapy for HIV] clinic…they put them in the GBV corner”. *(#6, F, Enrolled Midwife, Urban)

##### Availability of GBV screening tools

GBV screening tools are cues that may prompt clinicians to ask IPV-related questions because it recommends IPV screening for women who present with symptoms of abuse. Health facility-based IPV screening was facilitated by availability of both ANC registers which have a single yes/no IPV item and GBV incident reporting forms in ANC clinics provided by the Government of Uganda. One obstetrician stated that:

“*We use the ministry of health guidelines to screen and refer and attend to people like that.”. (*#24, M, OBGYN, Urban)

However, these screening tools are often not utilized because many providers are not aware that such screening tools exist as stated in the following excerpt:

*“The screening tools are there but they are not known much. I have seen a book of gender-based violence when I was in some health center III. That book [to document IPV] was there though it was not in use, but it was there. Maybe they could increase the SOPs [standard operating procedures], screening tools and even posters because I have seen the ones for family planning and people are well versed with family planning but not gender based violence”. *(#20, F, Enrolled Midwife, Urban)

Part of the reason that ANC providers may have been unaware of the screening tool is that they were not located in ANC clinics, but only in the specialized GBV clinics, which were not in every health center.

Many providers who were not aware of existing screening tools suggested that the characteristics for these tools should include having ‘few’ IPV items that can be administered quickly so as not to disrupt clinical activities or negatively impact waiting time in ANC clinics.

*“Not too bulky. If possible, let it take less than five minutes. You ask a few questions to know whether the husband is supportive during this pregnancy that will help her to open up. Some do not open up easily, so you may miss out those mothers but if it is a questionnaire, this will help to identify them very fast. A questionnaire that is specific to things at home, whether the husband is supportive or whether there is violence at home”. *(#2, F, Assistant Nursing Officer, Rural)

The existing GBV screening tool is brief, only one page long, but as mentioned, providers are not aware of it.

##### Modification of ANC cards by including IPV items

According to some healthcare providers, ANC cards, also called mothers’ passports, do not contain IPV items. Respondents proposed that the current version of ANC cards be modified to include questions probing IPV as a way of facilitating IPV screening during ANC clinic consultations. ANC cards are given to every pregnant women who attends ANC clinics, women keep the ANC cards home and return with them during every scheduled ANC visit. ANC cards are designed to prompt clinicians to elicit women’s medical, social, and family history as well as to document findings from clinical examination. ANC cards are also a way of monitoring women’s pregnancy and birth plans and to anticipate actions needed in case of complications or risk factors towards maternal or fetal health. However, the current version of ANC cards lacks specific items to elicit information about IPV. One provider suggested that including IPV items in ANC cards could act as cues that may increase IPV screening especially during busy clinic days as elaborated in this statement:

*“We need to come up with something very well organized and we need that information to be put in the mothers’ passport for antenatal. It can help us to ask such whenever you interact with pregnant mothers. If it is also included there, we can try to screen each and every mother who comes since we would have where to document [IPV].” *(#5, F, Assistant Nursing Officer, Urban)

According to one rural-based physician, IPV is usually detected during physical examination, a key opportunity to increase facility-based IPV detection especially during busy clinic days with a high patient-physician burden. However, ANC cards do not have prompts to elicit IPV as stated in the excerpt below.

*“The challenge is one, in rural areas, we were being overwhelmed by the number of patients. So, you just follow the assessment of the antenatal card, and antenatal card does not include domestic violence. Gender-based violence, it is not there. So, … normally we discover as I said during examination when you are one on one [with IPV survivors], that is when they can tell you.” (*#12, M, Medical Officer, Rural)

##### Inadequate physical space

Another reason for failing to conduct routine IPV screening is the lack of privacy within ANC clinics. Providers highlighted a need for separate rooms in ANC clinics in order to increase confidentiality as well as comfort during providers’ interactions with women. This suggests that the lack of privacy hinders IPV screening due to the sensitive nature of such issues asked especially during ANC appointment scheduling. Scheduling occurs during the initial ANC visit where detailed history taking and clinical evaluations are conducted including IPV screening. One midwife reported that IPV screening is not routinely conducted during ANC scheduling because of limited privacy.

*“When you want to explore issues concerning that [IPV], we need privacy. The best time to explore is the time of booking but the booking is done where there is no privacy”. *(#2, F, Assistant Nursing Officer, Rural)

Improving clinic infrastructure as a strategy to facilitate IPV screening was proposed by a rural-based ANC midwife stating that,

*“We need to get a private room. When we get a private room it will work, but unfortunately here our space is too small and the mother will not be willing to tell you in the open because even in the examination room where we examine mothers there are two beds so there is no one-to-one privacy”. *(#14, F, Enrolled Midwife, Rural)

#### Awareness and training to screen and respond to IPV

Healthcare providers identified two IPV screening barriers related to awareness and training to screen and respond to IPV, namely, lack of comprehensive GBV training and inadequate IPV screening knowledge. Healthcare providers expressed concern that they had not received training in GBV service provision. For example, some healthcare providers stated that many of them had not received any specific GBV training and that their knowledge on IPV screening was obtained mainly during their medical school training as described in this statement:

*“The challenge is that, personally, it is just because of the knowledge that I attained from school, but I have never gone through gender-based violence training. These workshops I have never. I just use the knowledge that I got from school, so we need training, continuous supervision and support”. *(#10, M, Enrolled midwife, Rural)

A few healthcare providers reported having received IPV training. However, they reported that when only a few people are trained, this reduces the likelihood that those who did not receive training will screen for IPV, letting the trained “experts” do it for them. This can serve to reduce screening overall if people who are trained in GBV leave the clinic as described by the participant below.

*“When you are training [on IPV], it is better to train everyone but then if you just train some groups of people, when they are transferred, they will just go with their knowledge. The ones who remain will not do the work because they will say let those who trained do that work.” *(#1, F, Enrolled Midwife, Rural)

A lack of IPV training meant that providers were often unsure of how to screen for IPV stating that:

*“One of the challenges is the knowledge gap. The right way to do the assessment, you may do it because you are a doctor, and you just ask your questions the way you think you should ask them. But is it the right way? So, there is that knowledge gap.”* (#9, M, Medical Officer, Rural)

Uganda clinical guidelines clearly emphasize the need to screen pregnant women for IPV in ANC clinics. While standardized screening tools for IPV exist in ANC clinics, many healthcare providers were unaware of them. They reported that having a standardized tool would help them ask about IPV in the “right way” as stated in the following excerpts:

*“There is no special tool which can help us or one can use to identify someone who is at risk of gender based violence. We surely do not have any tool to help us do the screening. So, it is from our observation that we develop high index of suspicion of gender-based violence.” *(#16, M, Medical Officer, Rural)

“*The way we screen, there is no form that will actually guide you in the screening”. *(#23, F, Enrolled Midwife, Urban)

#### Lack of awareness of the seriousness of IPV

##### Provider misperceptions

One rural-based physician echoed the practice of not routinely screening for IPV stating that some providers perceive IPV as a ‘home issue’, normalizing violence against women by assuming it does not exist unless the abuse is severe.

*“We do not normally screen them but when you are carrying out physical examination, that is when they can reveal those secrets. Gender-based violence, those are home issues unless it is severe that they can tell a health worker and that can happen only at the time of examination because we have not reached that level of screening them for gender-based violence”. *(#12, M, Medical Officer, Rural)

Another provider cited culture being an influence that keeps individuals and communities from disclosing IPV. This excerpt below asserts that beliefs held by communities, including some providers, that ‘outsiders’ should not be told about violence in their homes.

*“Yes, African culture … There is that saying … that the secrets for the family must remain in the family. They should not be taken to the outsiders, there is that saying.” *(#19, F, Enrolled Nurse, Urban)

#### Establishing trust

##### ***Provider initiated probing***

IPV is an emotional experience to survivors and, according to one obstetrician, this makes it uncomfortable for providers to ask and for patients to disclose.

“*The fact is that we have not tried so much to dig into intimate partner violence because one, it is not something that people bring up so easily. So, most people find it a little disturbing to start asking”. (*#4, M, OBGYN, Urban)

One urban based midwife stated that when healthcare workers initiate probing of women for IPV exposure, privacy is needed to develop rapport as described in the following quote:

*“Maybe you try to take that mother from the group [in triage and booking waiting areas], then try to ask and opens up. What is happening? Like are you ok with your husband? What is happening at home? If you try to ask questions, someone will come out and tell you what is happening”. *(#5, F, Assistant Nursing Officer, Urban)

Providers mentioned asking about IPV during physical exams if signs of abuse are apparent. Even with physical evidence of abuse, it can be difficult for women to open up as described by one rural-based medical doctor in this statement:

“*They tend to hide the information. Not until you really have the skills of probing, that is when you can get to know that this mother has a GBV problem”.* (#16, M, Medical Officer, Rural)

In fact, one urban-based provider stated that their probing is occasionally forceful, stating that some mothers are coerced to open up about spousal abuse experienced if they are not willing to open up.

*“Doctor looked at her and examined her and told her that tell us the truth because I did not believe this story. So, forcing her, she opened up and told us that she was beaten by the husband, the reason being the husband came in with another wife and he forced her to leave the bed and she was beaten seriously”. *(#3, F, Assistant Nursing Officer, Urban)

According to healthcare workers, the signs of physical and emotional IPV may be observable. Visible signs such as low moods suggestive of depression or bodily bruises may indicate potential physical trauma from spousal abuse. Both urban- and rural-based ANC providers described how high levels of IPV suspicion such as pre-screening practices for IPV may increase IPV detection:

*“From the way they present, this woman will come in with emotional distress which is not okay. So, from your observation and from the training you can assess that this mother is not okay. Sometimes you inquire what could have gone wrong, so that is when you may even know she had the gender-based violence”. *(#9, M, Medical Officer, Rural)

##### ***Rapport building***

Healthcare providers play an important role in GBV prevention and response in Uganda. The quality of patient-physician interactions contributes to trust building, whether clinicians screen for IPV or if pregnant women disclose spousal abuse as one urban-based midwife noted:

“*According to the relationship or the rapport you have made from the beginning with these mothers, some mothers open up and tell you what is happening at their home, … how the husband is treating her. So, she may tell you that my husband is like this [abusive] …, and normally people put trust in health workers. If you are really a friend, they can tell you everything because they know at times you can help”. *(#5, F, Assistant Nursing Officer, Rural)

One urban-based midwife explained why it is essential for healthcare providers to establish friendly relationships with patients prior to delving into asking women questions that may trigger strong emotions stating that,

*“I know that as you are going to approach this mother, create a relationship with her because she will never open up to you when you are not her friend. Why? You do not know her and she also does not know you but you want some important information, and when you become a friend, you create a rapport with this mother and she will pour out information”. *(#20, F, Enrolled Midwife, Urban)

However, the opportunity to establish confidence that abused women have in health care providers is often limited because of the high workload and time constraints facing ANC providers explaining:

*“After seeing that she has gained some confidence in you [ANC provider]…, she will come out to tell you what the real problem is”. *(#7, F, Assistant Nursing Officer, Rural)

##### Patient initiated disclosure

Some participants reported instances of unprompted IPV disclosure by pregnant women in ANC clinics even in the context of non-routine screening for IPV. Patients’ disclosure of abuse perpetrated by their intimate partners before healthcare providers ask women about IPV was reiterated by one OBGYN as described in the quote below:

*“There are mothers’ who tell you everything, even before we ask them if they are fine, whether their husbands beat them or slapped them.” (*#4, M, OBGYN, Urban)

## Discussion

The purpose of this study was to explore facilitators and barriers to routine screening for IPV by healthcare workers in rural and urban-based ANC clinics in Uganda. Our study revealed that many healthcare providers do not routinely screen for IPV in ANC clinics despite existing policy recommendations and clinical guidelines that emphasize the importance of identifying and responding to IPV in healthcare settings [17]. We also identified facilitators and barriers to routine IPV screening in ANC clinics in Uganda. The overarching emergent themes that may explain why some providers screen for IPV while some do not were resource availability, receipt of training in IPV, lack of awareness of IPV severity, and establishing trust.

Our findings revealed that many healthcare providers do not routinely screen for IPV among pregnant women attending ANC clinics. This was in line with prior research conducted by Kaye and colleagues which showed that several healthcare workers in an urban referral center in central Uganda neither knew how to, nor routinely screened for domestic violence [41]. Similarly, another study conducted among healthcare workers in Uganda, showed that some healthcare providers did not inquire about IPV exposure at all [42]. In our study, we further ascertained that some clinicians do not routinely screen for IPV because they did not know how to ask about IPV exposure. This may have been because of their lack of awareness of available IPV screening tools which could provide simple ways of asking about IPV. IPV screening also may not have occurred because many clinicians had not received any IPV-related training. Our findings were in line with prior studies conducted in Uganda which showed that some healthcare workers in an urban-based OBGYN unit lacked technical competence to provide optimal ANC care to IPV survivors [41]. Similarly, one study conducted in Uganda by Lawoko and colleagues demonstrated that medical doctors had lower self-efficacy for IPV screening when compared to other healthcare professionals such as midwives [29].

Women in HIC often prefer self-completed [43] or self-report screening tools [44] compared to face-to-face questioning by healthcare providers about spousal abuse. Such self-report tools are not commonly used in Uganda and may not be feasible due to low levels of literacy. Health facility-based screening tools for IPV in Uganda include: (i) GBV incident screening forms containing multiple IPV items [45] and (ii) ANC and family planning registers that have a single IPV item [46]. Although both IPV screening tools are core components of the GBV prevention and response strategy in Uganda [45], our study revealed that some healthcare providers do not ask pregnant women the single, yes/no item in ANC registers. This IPV item in ANC registers lacks corresponding questions which may explain why some providers in our study reported that they did not know how to ask attending women about IPV.

Although prior research has questioned use of ‘short’ IPV screening tools due to low validity in measuring IPV [47, 48], some studies found single-item IPV screening tools adequate [49], and valid for clinicians to elicit trauma history [50] as well as reliable in measuring IPV across diverse populations and cultures [51]. It is worth noting that IPV screening among socio-economically disadvantaged and disempowered women has a potential empowering effect on women who are and are not pregnant while also improving confidence and trust in patient-provider relationships [13]. Therefore, we argue that by ensuring that healthcare providers receive training on the use of brief, standardized IPV screening tools, IPV screening in ANC units may improve.

Reluctance to routinely screen for IPV or not screen at all can be attributed to work-related pressures arising from the high number of women attending ANC and understaffed ANC clinics. Healthcare workforce shortages remains a persistent and systemic challenge to health service delivery in Uganda. It is noteworthy that the extent of IPV screening is generally low in LMICs [24] including Uganda, when compared to relatively higher initial and repeat IPV screening rates during subsequent ANC visits in HICs [33]. Some of the factors that may account for higher rates of health facility-based IPV screening in HICs [52] include: more resources in terms of healthcare staffing and adequate patient-physician interaction time [53]. Since establishing trust often requires time, which is limited in busy ANC clinics, respondents in our study discussed several approaches that can be used to address the high patient-physician ratio in ANC clinics such as increasing provider staffing and task sharing. Increasing human resources for health and task sharing are health system strengthening strategies recommended for improved health service delivery in LMICs [54]. However, current staffing levels are still sub-optimal across the healthcare referral system in Uganda [55], which may contribute to inadequate routine IPV screening. Despite challenges of high patient-physician burden and low staffing levels, some providers in our study proposed two innovative mechanisms to improve routine IPV screening. First, they recommended creation of special GBV units in rural ANC clinics similar to those in urban healthcare settings in order to complement integrated ANC services aimed at preventing and responding to violence against women and girls. Second, focal persons could be added to the ANC health workforce of focal persons who are specially trained to focus on IPV service provision to perinatal IPV survivors attending ANC clinics.

Another resource related constraint to IPV screening was the lack of privacy in ANC units. In our study, healthcare providers who screened for IPV did so in examination rooms. Such rooms have some amount of privacy, which increases providers’ confidence to ask women questions about spousal abuse that are socio-culturally sensitive. In line with our findings, a prior health facility-based study conducted in Uganda demonstrated that the lack of an optimum environment, including inadequate privacy, contributes to provision of sub-optimal care to IPV survivors during ANC [41]. In line with the Uganda Clinical Guidelines [17], prior research recommends that healthcare workers create opportunities to routinely attend to women privately during ANC [56]. Physician consultations conducted in privacy reduces stress and anxiety which in turn increases chances of IPV disclosure among survivors. Partners’ presence in ANC clinics may deter providers from screening for IPV in cases where providers may suspect retaliatory abuse. Our study revealed more limited resources in rural areas, therefore, addressing space and staff shortages in rural settings should be a priority for strengthening the healthcare system which could then improve IPV screening.

Our study also found low levels of provider knowledge on perinatal IPV and how to screen for it. Several healthcare workers reported that they had not received any training specific to provision of IPV services with rural/urban differences observed. For example, in our study, only three out of ten providers who had received IPV training were rural based. Healthcare providers who had received IPV-related training acknowledged having low awareness on how to screen for IPV suggesting that ANC providers would benefit from comprehensive GBV trainings with emphasis on how to screen for IPV. Prior research in neighboring rural Kenya was in line with our findings demonstrating that healthcare workers lacked training on IPV related issues [57]. Prior studies recommend that healthcare worker training on IPV-related issues should place strong emphasis on the different forms of IPV [57], and that the number or duration of IPV trainings should be increased in order to increase the likelihood of recurrent or frequent IPV screening [58]. It is noteworthy that in a study conducted in rural US, while provider IPV knowledge did not predict IPV screening, the duration of IPV training and availability of institutional IPV protocols increased facility-based IPV screening. In addition, IPV training may change providers’ attitudes towards IPV as being a “home matter” as well as may challenge cultural norms that condone IPV.

Although some healthcare providers in our study had inadequate or no IPV training, routine IPV screening in ANC clinics was supported by availability of psychosocial counselors. Evidence supports the need for specialist domestic abuse clinicians as a useful resource to improve access [59], especially when providers experience challenging IPV survivor cases [60]. We further posit that providing IPV related training to healthcare providers is not enough and should be complemented with structural and system level GBV responses and prevention interventions [22, 61]. For example, trauma-informed care (TIC) are comprehensive, multi-level approaches adopted beyond healthcare facilities, that into account a patients’ life situation in order to realize the widespread impact of trauma, and paths to recovery from trauma [62]. TIC approaches respond by integrating trauma policies and practices in order to improve knowledge and recognition of trauma signs, as well as actively preventing re-traumatization [62]. Integrating TIC into routine clinical practice [63] could substantially increase the utility of GBV screening protocols and the subsequent practice of routine IPV screening in ANC clinics. Since most trauma healing can take place outside healthcare settings, trauma informed living environments need to be supported [64]. TIC approaches are survivor-centered and should be provided through creation of safe places, survivor empowerment, establishment of positive connections between service providers and survivors, and management of the emotional/psychological impact of trauma [65].

Diverse clinical specialties in ANC settings may increase the availability of different clinical skill sets, which aid in clinical decision making in IPV screening and detection if IPV screening tools and protocols are available. Since signs of non-physical IPV are subtle when compared to signs of physical IPV [6], healthcare workers may be more observant towards physical injury in women [66], as our study also revealed. Thus, it is important for providers to be familiar with non-physical forms of abuse in order to holistically probe for IPV in all their patients receiving ANC.

### Study strengths and limitations

We had the following study strengths. First, our study participants had different clinical qualifications and roles in the rural and urban ANC clinics which improves transferability of our findings to similar contexts. Secondly, data saturation was achieved from interviewing this diverse sample of twenty-eight ANC providers. Thirdly, we used a qualitative methodology, which are appropriate analytical approaches to explore healthcare providers persepectives on routine screening barriers and facilitators for IPV screening. Finally, credibility of our findings is likely due to member checking that was conducted. This study had some limitations. First, our study had limited diversity in terms of provider views about routine IPV screening in ANC, despite the larger sample of midwives and nurses who are the majority providers of ANC services. Secondly, our study findings may not be transferable to healthcare settings at referral levels other than HCIVs in the study areas. Thirdly, our findings may have been subject to potential response bias due to healthcare providers’ giving socially desirable views based on how they perceived other healthcare providers in the group would respond. Similarly, providers may have had reservations about disclosing their IPV screening practices out of anxiety of being blamed or possible punitive actions. Informed consent assured participants of the privacy and confidentiality of their responses. To address potential provider uneasiness, the interviewer took time to build rapport and respond to participant concerns or mistrust. Future research should consider triangulating views from a diverse group such as healthcare managers, administrators, or policymakers at different levels whose decisions may directly or indirectly impact routine screening in ANC clinics.

### Actionable points

- Increasing the practice of routine IPV screening may be achieved through improving rapport building, encouraging provider-initiated probing, and fostering patient-initiated disclosure.
- Health facility-based interventions are warranted to increase routine IPV screening. These include addressing healthcare workforce shortages and health infrastructure challenges to alleviate limited clinic interaction time, inadequate physical spaces to reduce privacy issues and support consult-room and triage-level IPV screening respectively.
- Training healthcare workers using trauma-informed care-based approaches and implementation in ANC settings [62, 67] may increase clinicians’ ability to realize the burden of trauma, recognize how all individuals involved with systems, organizations are affected by trauma and respond by putting this knowledge into practice.
- Providers posited the need to address safety concerns raised by some IPV survivors about retaliatory partner abuse. This highlights a need for safe ANC spaces/environment that encourages IPV disclosure and corresponding appropriate referrals.

## Conclusions

This research was conducted in order to provide key information to support health facility-based GBV responses and prevention efforts including innovative approaches to improve IPV screening practices of healthcare providers in ANC clinics. Implementation of initiatives to increase ANC-based IPV screening should focus on addressing resource availability, equitably increasing comprehensive, trauma-informed care based GBV training opportunities, raising awareness of IPV severity among healthcare providers and encouraging the establishment of trusting patient-clinician relationships. Advocacy efforts to fill sub-optimal healthcare workforce capacity and infrastructure to address effects of workforce shortages and privacy concerns at health facility level are underscored. Future research should test system-wide, innovative violence prevention and response approaches to increase IPV screening suggested by providers in this study.

## Supplementary Information

**Additional file 1.**

## Data Availability

The datasets used and/or analyzed during the current study are available from the corresponding author on reasonable request.

## Abbreviations

ANC: Antenatal care
CEmONC: Comprehensive Emergency Obstetric and Neonatal Care
GBV: Gender-Based Violence
HCIV: Health Center IV
HIC: High Income Countries
IPV: Intimate Partner Violence
IQR: Inter-Quartile Range
IRB: Institutional Review Board
KCCA: Kampala Capital City Authority
LMIC: Low and Middle Income Countries
MCW: Medical College of Wisconsin
MoGLSD: Ministry of Gender, Labor and Social Development
MoH: Ministry of Health
OBGYN: Obstetricians / Gynecologists
TASO: The AIDS Support Organization
UCG: Uganda Clinical Guidelines
UNCST: Uganda National Council of Science and Technology
WHO: World Health Organization
