# Supplementary material for: Facilitators and barriers to routine intimate partner violence screening in antenatal care settings in Uganda
Source: BMC Health Serv Res. 2022 Mar 2;22:283. doi: 10.1186/s12913-022-07669-0 (PMC8889632; doi:10.1186/s12913-022-07669-0)
Supplement: Supplementary file 1 — Additional file 1. [file 12913_2022_7669_MOESM1_ESM.doc]

**Appendix I: In-depth interview guide for healthcare providers**

1. Provider-related: Extent of prenatal IPV screening practices
2. Tell me a little bit about how you came to work with pregnant women and in this facility. What is your current position? What do you find most rewarding in your work? What do you find most challenging?
3. Tell me a little bit about the pregnant women you see during antenatal care. What is the average age of women attending antenatal care? Probe:
   - When do they usually come for their first appointments? How does this vary among women?
   - What do you think accounts for this variation? Tell me about men attending prenatal care with their pregnant spouses? How often does male partner ANC attendance occur?
4. What do you typically do at first antenatal visits? How about during the women’s follow-up ANC appointments? How many pregnant women do you see that you suspect suffer from IPV?
5. What would make you suspect that a woman is suffering from IPV? How would you ask a woman if she is suffering from IPV? Tell me about any expectations you have to conduct IPV screening during prenatal care? What suggestions do you make to increase identification of abused pregnant women? Probe: How may each suggestion be conducted effectively? How can each suggestion increase service delivery to abused pregnant women?
6. Tell me more about whether you screen of women for IPV. Generally, do you screen for IPV during prenatal visits. Probe why the reasons why providers decide to screen women for IPV. Why do they sometimes not probe women? Probe:
   - How do you assess pregnant women who may be vulnerable to partner abuse? In your opinion, is it routine practice to screen for IPV?
   - Do healthcare workers in the antenatal clinic usually screen pregnant women for Intimate partner violence?
   - How consistently do prenatal providers screen for IPV?
7. What has your experience been with women who suffer IPV? How about with pregnant women? How often do you assess pregnant women for IPV? Do you think that IPV screening should be conducted by all care providers interacting with pregnant women? How about by Village Health Teams? Why or why not?
8. Can you share any barriers to IPV screening during pregnancy?
9. What facilitates or impedes perinatal IPV screening in this unit?
10. Have you received training to conduct IPV screening? What type? What kind of trainings do you think you need? Are any trainings planned? What are your suggestions about how to improve the quality of prenatal care services? Probe:
    - How does the daily physician workload affect the ability to detect IPV?
    - Any competing priorities?
    - Suggestions to improve prenatal IPV screening
11. Referral and follow-up practices
12. Do you make any referrals when women screen positive for IPV? If yes, where are referrals

usually made and for what services do you refer these women? Probe: for social or legal services?

1. Do you follow-up these women who screen positive for IPV? How and who conducts these

follow-ups? What is done during these follow-ups?

1. What could be done to improve screening for IPV by care providers? How about by VHTs? What

could be done to better their quality of life, health and the effectiveness of treatment?

1. How are non-prenatal care services such as mental health care provided to pregnant women with mental health needs?
2. Can you suggest any recommendations on how to address IPV? Probe:
   - IPV detection of IPV in healthcare settings or community
   - Suggestions about where referrals for support services should be made.
3. Do you think there is a gap or discordance between usual screening practice for IPV by clinicians

and guideline recommendations?

1. Partner-related
2. What are some of the reasons that a pregnant woman would not report abuse by their partner?

How about reasons why women who are not pregnant may not report cases of intimate partner violence?

1. What suggestions would who propose on how to increase reporting of intimate partner violence

by women? What do you think are the barriers to reporting intimate partner violence by women? What suggestions do you propose for safety to women who make notifications of intimate partner violence? Probe:

- Recommendations on how to increase notifications and safety among intimate partner violence survivors who report intimate partner violence
- Should intimate partner violence be routinely reported in HMIS?

1. Organizational
2. Does this health facility possess guidelines on IPV screening and referrals?
3. How does the clinic implement the clinical guidelines on IPV screening?
4. What are the health facility’s monitoring and reporting strategies for IPV using the Health

Management Information Systems (HMIS)?

1. Tell me about the Ministry of Health policy on IPV screening in women and men? What are the

clinical guidelines on IPV screening? Probe:

Probe: What is the policy or guidelines on IPV screening during pregnancy? What is the usual practice for IPV screening during pregnancy? How easy or difficult are these guidelines to follow? Probe:

- How consistent is IPV screening in this facility? In this facility and clinical practice, is IPV
- screening common? If yes, what encourages consistent screening? If no, why is IPV screening not routinely practiced?
- What challenges do you experience in conducting IPV assessments during prenatal care? What would you recommend to improve IPV screening during pregnancy by care providers?
